# Advancements in Developing Strategies for Sterilizing and Functional HIV Cures

**DOI:** 10.1155/2017/6096134

**Published:** 2017-04-26

**Authors:** Wei Xu, Haoyang Li, Qian Wang, Chen Hua, Hanzhen Zhang, Weihua Li, Shibo Jiang, Lu Lu

**Affiliations:** ^1^Key Laboratory of Medical Molecular Virology of Ministries of Education and Health, School of Basic Medical Sciences and Shanghai Public Health Clinical Center, Fudan University, Shanghai 200032, China; ^2^Key Laboratory of Reproduction Regulation of NPFPC, SIPPR, IRD, Fudan University, Shanghai 200032, China; ^3^Lindsley F. Kimball Research Institute, New York Blood Center, New York, NY 10065, USA

## Abstract

Combined antiretroviral therapy (cART) has been successful in prolonging lifespan and reducing mortality of patients infected with human immunodeficiency virus (HIV). However, the eradication of latent HIV reservoirs remains a challenge for curing HIV infection (HIV cure) because of HIV latency in primary memory CD4^+^ T cells. Currently, two types of HIV cures are in development: a “sterilizing cure” and a “functional cure.” A sterilizing cure refers to the complete elimination of replication-competent proviruses in the body, while a functional cure refers to the long-term control of HIV replication without treatment. Based on these concepts, significant progress has been made in different areas. This review focuses on recent advancements and future prospects for HIV cures.

## 1. Introduction

Combined antiretroviral therapy (cART) has enabled the sustained control of viremia in virtually all human immunodeficiency virus (HIV) patients. It has prolonged lifespan, improved quality of life, and transformed HIV infection from a fatal disease into a chronic infectious disease [1–3]. However, individuals on cART require lifelong adherence, and withdrawal of the therapeutic regimens inevitably leads to rebound of HIV replication. In addition, long-term medication may increase the risk of adverse reactions, such as immune system disorders, nervous system disorders, and increase of viral reservoirs. Therefore, new theory and methods are urgently needed for the development of an effective HIV cure.

The key obstacle to an HIV cure is latent HIV reservoirs, which are mainly composed of resting memory CD4^+^ T cells in the early stages of HIV infection [4, 5]. During transcription of the provirus DNA is inhibited, thereby allowing the provirus to evade clearance by the host immune system. Although cART is directed against cells that replicate HIV, it has no effect on cells carrying latent HIV reservoirs, demonstrating the ineffectiveness of cART as an HIV cure.

Two types of HIV cures are under development: the “sterilizing cure” and the “functional cure.” A sterilizing cure refers to the complete elimination of replication-competent proviruses. The famous “Berlin patient” represents one successful case of a sterilizing cure. Timothy Brown, the so-called Berlin patient, positive for both HIV and acute myeloid leukemia (AML), received two stem cell transplants from a donor homozygous for the CCR5delta32 mutation. The CCR5delta32 mutation stem cell is a kind of CCR5-deficient cell, which renders cells highly resistant to HIV-1 infection. Eight years later, he appears to be free of both HIV and AML [6]. However, it is very difficult to find donors with human leukocyte antigens (HLA) identical to those of recipients for CCR5 Delta32/Delta32 stem cell transplantation, while the mortality rate of transplant surgery is up to 30%. Thus, this treatment model is difficult to reproduce. However, other strategies to carry out an effective sterilizing HIV cure are under development, such as genome editing, gene therapy, and shock and kill [7, 8].

Functional cure refers to the long-term control of HIV replication, which involves maintaining a normal CD4^+^ T cell count and HIV replication below a detectable level [9]. “HIV controllers” are considered to be those patients whose HIV RNA is kept below the clinical baseline for a long period without cART. Studies on “HIV controllers” are expected to provide important clues for the development of therapies or strategies for functional HIV cure, such as therapeutic vaccines and vector-mediated gene transfer therapy [10, 11]. Moreover, the human genome has integrated a large number of retrotransposon sequences over the course of evolution, and HIV may coexist with humans if it is restricted. From this perspective, the functional cure is as important as the sterilizing cure. This article will review the advancements in developing strategies for both sterilizing and functional HIV cures.

## 2. Strategies for Sterilizing HIV Cure

### 2.1. Gene Therapy to Eradicate HIV Reservoirs

**Figure 1:**
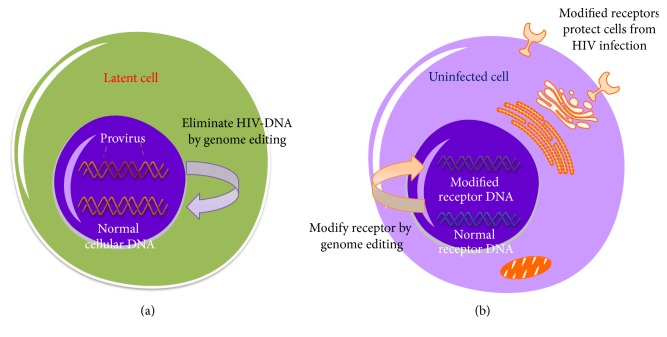
Two major strategies for HIV cure by using genome editing. (a) Gene therapy strategies to eradicate HIV reservoirs. Using ZFN, TALENS, or CRISPR to eliminate the HIV provirus in latent cells. (b) Gene therapy strategies to prevent susceptible cells from HIV infection. Using gene editing to modify the receptor of susceptible cells and protect them from HIV infection.

Three major genome editing technologies have been used to eliminate the HIV provirus, including Zinc-finger nuclease (ZFN) technology, the effects of transcription activator-like effector (TALENS), and clustered normal interspaced short palindromic repeat (CRISPR) and CRISPR-associated protein 9 (CRISPR-Cas9) technologies [12–14]. In contrast to normal cells, HIV reservoir cells harbor a latent reservoir of HIV proviruses with the potential for replication. Therefore, “targeted elimination” of these cells will reduce their ability to create HIV viral offspring. Accordingly, some researchers use genome editing technologies to mutate the target fragments of HIV proviruses in latent reservoir cells (Figure 1(a)).

In 2011, Wayengera used ZFN technology to abrogate the function of the* pol* gene. However, the modification of the coding sequence could not completely silence the HIV provirus, and the unmodified viral genes were still expressed under the effect of long terminal repeat (LTR) [15]. Qu et al. then presented a possible alternative therapeutic approach by using specially designed zinc-finger nucleases (ZFNs) to target a sequence within the LTR to directly mediate a deletion of the HIV provirus from the HIV-integrated human T cell genome [16]. The target sequence was conserved across all HIV clades making it suitable for a variety of HIV genotypes. Moreover, they found that effective excision of LTR could clear full-length HIV-1 proviral DNA. In their experiment, they observed that the frequency of excision was 45.9% in infected human cell lines after treatment [17]. In 2014, the team used the LTR U3 region as a target of ZFN technology and successfully cleared about 30% of HIV proviruses from infected cells.

One major challenge for both ZFN and TALENs is the expense and labor involved. A new genome editing tool CRISPR/Cas9 has recently entered the spotlight. From 2013 to 2014, two groups edited the integrated LTR on the cell genome using the CRISPR/Cas9 system, respectively, and succeeded in suppressing LTR-controlled gene transcription [18]. The CRISPR/Cas9 system mutates not only HIV DNA in T cells but also some other HIV reservoir cells, such as microglial cells and monocytes-macrophages. Furthermore, CRISPR/Cas9 was verified to eliminate multiple HIV DNA copies [18]. Studies show the potential application of the CRISPR/Cas9 system in curing HIV infection. By making site-specific DNA double-stranded breaks, the CRISPR/Cas9 system is also a unique antiviral defense system against foreign plasmids and viruses. A recent study reported that the CRISPR/Cas9 system interrupted the latently integrated viral genome, thereby forming a barrier against viral infection, suggesting the CRISPR/Cas9 system as a new therapeutic strategy [19].

Another study revealed that the CRISPR/Cas9 gene editing technique could prevent the HIV virus from infecting T cells. A CRISPR-Cas9 genome editor introduced a mutation in embryos that caused dysfunction of the CCR5 gene [20]. Meanwhile, the Kaminski team found that this gene editing system could remove all HIV-1 proviral DNA copies from latently infected human CD4^+^ T cells by using a convenient human T-lymphocytic cell culture model and CD4^+^ T cells obtained from HIV-1^+^ patients. In this study, it was confirmed that this technique not only removed the proviral DNA from T cells, but also subsequently protected them from new HIV-1 infection. This research team also addressed the issue of off-target effects and toxicity [21]. In 2015, Strong et al. used transcription activator-like effector nucleases (TALENs) to target the LTR of the HIV-1 provirus, thus preventing the expression of LTR downstream genes [17].

Although the gene therapy strategy for HIV provirus DNA is a direct method for eliminating the HIV reservoir, two challenges remain. First, all of these studies were based on in vitro experimentation or nonhuman primates, but no human experimental data, essentially because no efficient vector could transfer these elements to the targeted gene. Without specific targeted introduction, the editing effect will be minimized since all cells can potentially take up these elements. Second, genome editing technologies have varying degrees of “off-target” effects, and although no cytotoxicity and cell death have been observed in the primary cells, this potential risk cannot be completely ruled out.

### 2.2. Gene Therapy to Prevent Susceptible Cells from HIV Infection

Successfully curing HIV infection in the “Berlin patient” case resulted from the transplantation of CCR5 Delta32/Delta32 stem cells [22]. Thus, if latent HIV-infected cells lose susceptibility, newly infected cells will be limited, and a “clean” population of CD4^+^ T cells will be established [23, 24]. Inspired by the “Berlin patient,” Perez et al. used engineered zinc-finger nucleases (ZFNs) to disrupt endogenous CCR5 in primary human CD4^+^ T cells, and 50% of CCR5 alleles of these cells were mutated (Figure 1(b)). The modified CCR5 genotype could be inherited in the process of amplification of CD4^+^ T cells in vitro. The genetic mutation of CCR5 provided stable and heritable protection against HIV infection in a NOG model of HIV infection such that engrafted, ZFN-modified mice had lower viral loads than mice with wild-type CD4^+^ T cells [25]. Tebas et al. enrolled 12 patients with chronic HIV infection while receiving highly active antiretroviral therapy (HAART) [26]. Six of the patients were infused with 10 billion CD4^+^ T cells for four weeks, followed by withdrawal of cART. Although HIV titers rebounded after cART withdrawal, the numbers of CD4^+^ T cells in peripheral blood of all patients were significantly higher than those in the control group. Additionally, the viral titers in most patients continued to decrease after reaching a peak at 16 weeks, and the HIV RNA level of one treated patient became undetectable at the endpoint of the test. These results suggested that improving the efficiency of ZFN editing and obtaining double mutations of CCR5 alleles might significantly improve the therapeutic effect. Only one patient had fever, chills, joint pain, and back pain after infusion [26].

Recently, Badia et al. reported a new strategy targeting the region of 32-deletion polymorphism in the CCR5 gene (CCR5Δ32), which mimicked the naturally occurring CCR5Δ32 polymorphism in the “Berlin patient,” to further confirm the safety of this therapy [27]. Based on the reconstruction of CCR5 therapy in CD4^+^ T cells, some patients could be withdrawn from cART after a certain period, seeming to indicate a dawn of functional cure. The challenge of this therapy is that the adoptively mature CD4^+^ T cells have limited replicative potential. Thus, the depletion of infusing CD4^+^ T cells in peripheral blood led to virus rebound. Therefore, some researchers are studying how to make HIV-infected individuals continue to produce CCR5-modified T cells. For instance, Holt et al. modified CD34^+^ human hematopoietic stem/progenitor cells (HSPCs) by ZFN targeting to CCR5 in vivo and transplanted the modified cells into nonobese diabetic/severe combined immunodeficient/interleukin 2r*γ*null (NOD/SCID/IL2r*γ*null; NSG) mice, while the control mouse group received untreated HSPCs. When challenged with a CCR5-tropic HIV virus, the control mouse group showed profound CD4^+^ T cell loss [28]. In a recent report, Li et al. mobilized CCR5 gene expression in HSPCs using a recombinant adenoviral vector encoding a CCR5-specific pair of zinc-finger nucleases. Hematopoietic stem cell transformation and autologous transplantation are expected to achieve a viable functional cure [29]. Theoretically, mobilizing CCR5 gene expression renders HIV-susceptible cells less susceptible to HIV infection. Modified hematopoietic stem cells can continuously produce anti-HIV cells, including HIV target cells such as CD4^+^ T lymphocytes and macrophages. However, hematopoietic stem cells can easily lose their differentiation ability during in vitro culture. The main challenges for the technology are how to efficiently, accurately, and securely deliver stem cells into bone marrow.

On the other hand, it is still debatable whether the modification of CCR5 can inhibit HIV replication of C-X-C chemokine receptor type 4 (CXCR4) tropism in vivo [29]. In order to avoid preexisting virus strains that use CXCR4 rebound, Didigu et al. used zinc-finger nucleases (ZFNs) to simultaneously inactivate the CCR5 and CXCR4 genes. They observed that the modified cells could inhibit virus replication for both CCR5 and CXCR4 tropism virus in humanized mouse models [30]. In general, the result of the “Berlin patient” indicates that the strategy of receptor gene editing is viable to some degree. And yet it is far from preclinical or clinical trials. Moreover, transformation, amplification, and reinfusion of T cells or hematopoietic stem cells in vitro are very complicated processes and treatment cost is high, making implementation uncertain. Although a number of Phase I clinical trials have been conducted, this treatment regime is still out of reach for HIV-infected persons in developing countries. In addition, some studies suggest that modifications of CCR5 may reduce the ability of the immune system to defend against pathogens. Also, whether CXCR4 modification affects its normal function requires further study [31].

### 2.3. Shock and Kill Strategy

Complete removal of HIV-1 viruses must eliminate infectious virions inside and outside the cell but also eliminate the provirus gene hidden in the infected cells and latent reservoirs. Using clinical drugs, good results have been achieved for the virus outside the cell. However, a true HIV cure will only result from the removal of HIV latent cells, which are those cells that remain “silent” or nonproductive after integration of HIV-1 genomes. With the exception of the HIV latent genome, these cells show little difference from normal cells, making them difficult to discover and eradicate. However, HIV-1 proviruses in latent cells can be reactivated under certain conditions, resulting in a viral rebound after drug withdrawal [32]. Importantly, latent cells have a long half-life in vivo. Studies have shown that the half-life of latent cells is about 43.9 months in vivo. Even in patients treated with HAART, a period of 73 years is required to completely remove latent cells, meaning that we cannot cure HIV with the existing drugs in a typical lifetime [33]. In order to cure HIV, the research community is now focused on approaches to clear persistent HIV infection, also known as the “shock and kill” strategy [34]. This approach activates latent cells and then uses existing HIV drugs to inhibit new generation of HIV infectious viruses. Two kinds of drugs are required. One is considered a “shock agent” to activate latent HIV cells, and the other is a “kill agent,” which blocks the reactivation of the latent virus and protects cells from forming new latency.

#### 2.3.1. “Shock” Agents

Several functional agents have the potential to revert HIV-1 latency, including (a) histone deacetylase (HDAC) inhibitors, (b) bromodomain and extraterminal (BET) inhibitors, (c) DNA methyltransferase (DNMT) inhibitors, (d) protein kinase C (PKC) activators, (e) cytokines and chemokines, and (f) unclassified agents [35, 36].

Among the “Shock” agents, HDAC inhibitors have been widely studied in the past decade. HDAC inhibitors, such as valproic acid (VPA), butyric acid, trichostatin A, vorinostat/SAHA, panobinostat, entinostat, givinostat, and romidepsin, have been well characterized, both in vivo and in vitro (Table 1). VPA was the first HDAC inhibitor to reach clinical trials. However, it was nonspecific, and limited effects were observed when activating latent cells [37]. Vorinostat is the first HDAC inhibitor approved by the FDA for the treatment of cutaneous T cell lymphoma. Reporting their findings in* Nature* in 2012, Archin et al. found that a single dose of vorinostat was effective in disrupting HIV-1 latency in patients with long-term treatment of HAART and significantly increasing RNA expression (mean increase 4.8-fold) [38]. Moreover, their finding showed the feasibility of using HDAC inhibitors as a “shock agent” to activate HIV latency in cells. HDAC inhibitors have several advantages over other latency reactivators in that most have been used as anticancer therapies whose pharmacological and toxicological properties are quite clear. Valproic acid (VPA), butyric acid, vorinostat, trichostatin A, and panobinostat have been approved by the FDA for the treatment of cancer. Some minor or self-limiting adverse effects have been reported for HDAC inhibitors, including nausea/vomiting and fatigue [39].

In addition to HDAC inhibitors, BET inhibitors, DNMT inhibitors, PKC activators, cytokines and chemokines, and unclassified agents have been shown to reverse HIV-1 latency. The representative agents for each groups are listed as follows: JQ1 is a BET inhibitor which binds to bromodomains and inhibits the function of Brd4 [40]. Moreover, JQ1 was found to synergize with other agents, such as HDAC inhibitors, to reactivate HIV from latency [41]. Currently, a thienotriazolodiazepine compound, OTX015, was reported to effectively reactivate latent provirus in a model of HIV latency with EC_50_ value much lower than JQ1 [42]. Likewise, 5-aza-2′deoxycytidine (Aza-CdR), a DNA methyltransferase inhibitor, which has been approved by FDA for the treatment of myelodysplastic syndrome, has a synergistic effect with NF-*κ*B activators [43]. Recently, a new protein kinase C (PKC) activator, Ingenol B, has been used to reactivate HIV latency in vitro. Because Ingenol B has minimal cell toxicity, it is more potent than SAHA and JQ1 [44]. Several novel unclassified agents, such as ZF-VP64 and TALE1-VP64, can also significantly reactivate HIV-1 expression from latently infected cells [45, 46]. ZF-VP64, a zinc-finger transcription factor, is composed of designer zinc-finger proteins and the transcriptional activation domain VP64. Similar to ZF-VP64, TALE1-VP64 consists of a DNA-binding domain and the herpes simplex virus-based transcriptional activator VP64 domain. Both of these are able to specifically and effectively reactivate latent HIV-1 transcription in vitro [45, 46]. Importantly, combinations of reagents with different mechanisms of action have shown efficacy. For example, the nucleoside transport inhibitor dilazep, HDAC inhibitor MC1293, and arsenic trioxide (As2O3) all synergistically reactivate latent HIV-1 with other activators, providing a new HIV latency reactivation strategy [47–49] (Table 1).

#### 2.3.2. “Kill” Agents

Besides current clinical anti-HIV drugs, such as cocktail therapy, “kill” agents can be used to clear virus-producing cells. These agents include therapeutic vaccines, broadly neutralizing antibodies (bNAbs), and nonneutralizing antibodies.

Studies have shown that while HAART suppresses HIV replication, it cannot eliminate the latent virus. Moreover, HAART also fails to kill infected CD4^+^ T cells after latent viruses are reactivated. Therefore, even though new virus generation can be inhibited by HAART, reactivated latent HIV virus can still survive to produce new virus, and an HIV cure will still not be achieved.

Therefore, therapeutic vaccines have been proposed with the hope of using them to induce HIV-specific cytotoxic T lymphocytes (CTL) to kill the reactivated latent cells. HIV-1-specific CTLs which could be stimulated by specific antigens are a major component of viral eradication [50]. In addition, these can be obtained by genetic engineering methods in vitro, such as transforming peripheral CD8 lymphocytes into HIV-1-specific CTLs by using lentiviral vectors encoding an HIV-1-specific TCR (T cell receptor, TCR) [51]. However, recent studies have shown that it is very difficult to induce a large number of highly efficient and specific CTLs in patients with severe HIV AIDS in vivo and that its effect on the killing and scavenging of latent library cells after activation is still controversial [52, 53]. Most patients receiving cART in the chronic phase have a presence of CTL immune escape mutant. In fact, these mutant cells are the reason for virus rebound after cART. This phenomenon also suggests that most latent cells have a certain ability to escape CTL before activation [52]. Therefore, the strategy of inducing specific CTLs by therapeutic vaccine and clearing the activated latent library cells remains a challenge.

Recently, Halper-Stromberg et al. found that combinations of inducers and bNAbs could interfere with the establishment of a silent reservoir in humanized mice without viral rebounding after drug withdrawal. The results showed that 57% of the hu-mice treated with antibodies plus combination inducers failed to rebound by the terminal point [54]. This research shows some promise for this therapy in the activation (shock) step, as well as the importance of the application of antibodies in the eradication (kill) step [55–57]. At the same time, the study also found that the Fc region of neutralizing antibody plays an indispensable role in HIV eradication, possibly through Fc-mediated mechanisms, such as antibody-dependent cellular cytotoxicity (ADCC). For instance, activated HIV cell-surface expression of the HIV envelope protein Env can be recognized by bNAbs. Also, Fc segment binding to Fc receptors on NK cells can kill latent cells. These results suggest that Fc function and Fc-mediated ADCC can be used to design drugs able to kill activated HIV latent cells. Subsequently, several studies have shown that ADCC may play an important role in killing the reactivated latent cells [58]. In addition, their results showed that about 40% of mice had viral rebound after drug withdrawal, suggesting that the use of neutralizing antibodies alone would face limitations but that further modification and optimization would enhance their ability to kill latent cells and eliminate the latent reservoir.

### 2.4. Strategies with Yet-to-Be Known Mechanisms

While HIV neutralizing antibodies can specifically neutralize HIV infection or inactivate HIV-infected cells, some non-anti-HIV antibodies may also be involved in the clearance of HIV-infected cells through some yet-to-be known mechanisms. A recent study showed that use of a monoclonal antibody (mAb) against *α*_4_*β*_7_ integrin in combination with ART resulted in the maintenance of low viral loads and normal CD4^+^ T cell counts in simian immunodeficiency virus- (SIV-) infected rhesus macaques for more than 9 months after the withdrawal of ART [59]. Anti-*α*_4_*β*_7_ mAb contributes to controlling viremia and reconstituting immune systems although the mechanism is unknown. This finding offers a new hypothesis for a cure that will be tested in the future. Interestingly, although anti-*α*_4_*β*_7_ mAb does not have SIV neutralizing activity, it significantly promotes gp120 V2-specific antibody responses, which played a key role in reducing the rate of HIV infection in RV144 clinical trials [60]. Therefore, we proposed that an HIV prophylactic and therapeutic vaccine could be designed by combining anti-*α*_4_*β*_7_ mAb with a V2-containing antigen in gp120 for induction of strong V2-specific antibody responses against HIV-1 infection because (1) both V2-specific B cell receptor (V2-BCR) and *α*_4_*β*_7_ integrin are expressed on B cells; (2) binding of V2-containing antigen (such as gp120) to *α*_4_*β*_7_ integrin would block its binding to V2-BCR; and (3) blocking V2-containing antigen binding to *α*_4_*β*_7_ integrin by anti-*α*_4_*β*_7_ mAb would allow the V2-containing antigen to bind with V2-BCR on B cells to induce V2-specific antibody responses against HIV-1 infection [61].

## 3. Strategies for Functional Cure

### 3.1. Vector-Mediated Gene Transfer Therapy to Achieve Functional Cure

Because existing HIV prophylactic vaccines fail to stimulate the immune system to generate bNAbs, researchers administered gene vectors, named vectored immunoprophylaxis (VIP), to animals to achieve long-term expression of bNAbs in the treated animals. The adeno-associated virus (AAV) is the most common vector; its safety and efficiency of gene expression have been fully verified [62–64]. Balazs' group injected AAV serotype 8 (AAV8) with a bNAb gene into humanized mice [65]. Later, Horwitz et al. injected 2.5 × 10^11^ genomic copies of bNAb 10-1074-expressing AAV into the muscles of the HIV-infected humanized mice pretreated with both cART and immunotherapies [66]. Results showed that nearly half of the mice exhibited a suppressed viral load after cART withdrawal. The same group has tested another bNAb, 3BNC117, in simian human immunodeficiency virus- (SHIV-) infected macaques and HIV-1-infected patients. They found that a single injection of 3BNC117 resulted in long-term (~13 weeks) protection of macaques against repeated SHIV challenges [67]. More interestingly, 3BNC117 infusion could significantly improve neutralizing responses to heterologous tier 2 viruses in almost all study HIV-1-infected patients tested, suggesting that 3BNC117-mediated immunotherapy enhances host humoral immunity against HIV-1 [67–70].

Although escape mutations occurred during the bNAb treatment, these studies have proven that the “gene transplant” strategy of bNAbs could become an acceptable substitute for cART for the first time [65–67]. The research also points a direction for the “gene transplant” strategy against HIV, which involves introducing multiple existing bNAbs into the body at the same time or finding and using super bNAbs targeting all genotypes and mutants to overcome strain diversity and escape mutants. The former requires gene transplantation many times over and may not necessarily prevent HIV escape mutants in the long term [65]. In contrast, the latter is more ideal, but it is difficult to find the super bNAbs that can avoid escape mutation and neutralize all HIV isolates, which is the key obstacle to prevention and a functional cure of HIV [67].

New research is expected to solve this problem. Gardner et al. created an immunoadhesin form of CD4, named eCD4-Ig, which is comprised of CD4-Ig and CCR5mim1 fused to the human IgG1 Fc domain [63]. In eCD4-Ig, the sulfopeptide CCR5mim1 was fused to the C terminus of CD4-Ig. This combination prohibits gp120 from interacting with CD4 and the coreceptor-binding site on the surface of the cells; thus, it functionally neutralizes the virus. eCD4-Ig exhibits high affinity and neutralizing ability for all isolates of HIV-1, as well as mutant strains resistant to other neutralizing antibodies. It can even neutralize HIV-2 and SIV, whose neutralizing effect has previously been beyond all bNAbs. All genotypes of HIV need to interact with the CD4 molecule and coreceptor molecule to enter cells. Therefore, it was estimated that the HIV-resistant mutants of eCD4-Ig were less prone to occur. On the other hand, eCD4-Ig had a stronger ability to mediate ADCC than the broadly neutralizing antibody b12. To reduce the immunogenicity of eCD4-Ig in the animal experiments, Gardner et al. substituted the D1D2 domain of CD4 from humans into the CD4-D1D2 of rhesus macaques to get rh-eCD4-Ig. In the six challenge experiments of SHIV during the period, all rhesus macaques in the treatment group were fully protected. The aim of the challenge experiments was to prove directly that eCD4-Ig had been synthesized and was able to prevent new infection. Applying the gene transplant of eCD4-Ig to HIV-infected individuals was expected to improve performance better than the gene transplant of existing bNAbs since eCD4-Ig can mediate ADCC with broadly efficient neutralization.

In recent years, many immunoadhesins that share functions in common with eCD4-Ig have been created. 2DLT, which was created by the combination of the D1D2 domain of the CD4 molecule in the human body and polypeptide HIV entry inhibitor T1144, inhibits and inactivates multiple clades of HIV isolates with high efficiency [71]. This protein is also considered an HIV protein inactivator because it was proved to inactivate free HIV virions. Furthermore, the 2DLT protein bound specifically with gp120 and gp41 on the surface of HIV-1 virions and destabilized the gp41 prehairpin fusion intermediate induced by CD4 domains, thereby significantly reducing sCD4-mediated enhancing effects on HIV-1 infection and providing a new solution for CD4-induced infection. Further study also showed that combining 2DLT with clinically used anti-HIV drugs, including HIV entry inhibitors, nucleoside and nonnucleoside reverse-transcriptase inhibitors (NRTIs and NNRTIs), and protease inhibitors, exhibited synergistic anti-HIV-1 activity against infection by divergent HIV-1 strains, including those resistant to NRTIs and NNRTIs, indicating that 2DLT has a potential to be further developed as a novel anti-HIV-1 drug for functional HIV cure [72].

Another example is an engineered bispecific multivalent protein, 4Dm2m, which contains 4 copies of mD1.22, the modified D1 domain in the CD4 molecule targeting the CD4-binding site (CD4bs), and 2 copies of m36.4, a human neutralizing mAb targeting the coreceptor-binding site (CoRbs), also known as CD4-induced site (CD4i), on gp120 [73]. Compared with D1D2, mD1.22 has higher affinity and specificity towards the CD4 binding site in gp120. Based on its origin from part of the D1 domain on the CD4 molecule, adverse reactions are expected to be lower in comparison with other immunoadhesins on the D1 and D2 domains [71, 74]. 4Dm2m exhibits highly potent antiviral activity against a broad spectrum of HIV-1 strains and high stability [74]. Unlike eCD4-Ig, 4Dm2m does not need several enzymes to activate it in vivo, making 4Dm2m easier to adopt for gene transplant strategies.

Recent findings showed that therapeutic vaccines and cytokines could enhance HIV-specific cell-mediated immune responses, but with only partial antiviral effects [75]. A primary difficulty in modifying permissive cells for the strategies mentioned above lies in their “personalization” in that permissive cells of each patient must be collected and modified instead of manufacturing and using the same type of drug, as in traditional therapies. Consequently, researchers asked how one type of drug could be applied to prevent permissive cells from being infected by the virus. The bNAbs and HIV entry inhibitors or inactivators are the best candidates. Since input antibodies and entry inhibitors have a short half-life in vivo, requiring long-term injection, the treatment cannot be regarded as an HIV cure strategy. However, vector-mediated gene transfer does enable exogenous proteins, such as antibodies, stably expressed for a prolonged period to be widely applied in HIV prevention and treatment for HIV cure. The approach involves the expression of adequate proteins that can inhibit viral infection stably in the long term, such as bNAbs, preventing cells from being infected by the virus so that the number of normal CD4^+^ T cells remains stable. The immunoadhesins discussed above are broader than bNAbs and unlikely to trigger HIV escape mutants. However, they are artificial and need long-term expression in vivo to realize the functional cure of HIV infection, making it necessary to further study their immunogenicity and adverse reactions.

### 3.2. New Ideas on Therapeutic Vaccines

Apart from the curative strategies introduced above, therapeutic vaccines have been continuously designed, essentially because training the immune system with vaccines is safer, cheaper, and more efficient than any other medication or treatments. The common design routes of therapeutic vaccines are focused on how to activate the immune system effectively, improving its identification and eradication of HIV and HIV-infected cells. Nevertheless, the strategy of using vaccines has thus far failed to enable the human body to generate enough effective bNAbs and CTLs. Therefore, immunogen design needs the implementation of unconventional strategies. One example is the HIV functional protein Tat (transactivator of transcription), which is an essential protein regulating viral replication and activation of the latent reservoir. It is also an important protein toxin in the process of HIV infection. In recent years, vaccine experiments based on Tat as an immunogen have reached phase II clinical trials [76–79]. Research of the Tat vaccine paves the way for using relatively conserved HIV “nonstructural proteins” as a therapeutic vaccine antigen [13].

The N-terminal heptad repeat (NHR) of the HIV Env protein gp41 is a conserved sequence of the HIV envelope. On the surface of HIV, NHR is covered by gp120 and is exposed only before membrane fusion. The process proceeds rapidly, and the space between the virus membrane and cell membrane is small. This limitation of time and space makes it difficult for an IgG antibody (MW = ~150 Kd) to access to and bind with NHR [80], although some well-designed immunogen containing the epitope of NHR could induce some antibodies in animals with moderate neutralizing activity [81, 82]. Nevertheless, some NHR-specific antibodies become neutralizing when incubated at the suboptimal temperature. This finding suggests that, at lower incubation temperature, the transition from the prehairpin intermediate state to the posthairpin fusion state can be slowed down, making it possible for IgG to access to and bind with the gp41 NHR to block HIV-1 fusion with the target cell membranes [83].

Recently, Wang et al. used an N63 peptide derived from the NHR sequence to immunize animals and tested the IgG antibody isolated from the sera of the immunized against HIV-1 infection. They found that the IgG antibody had no HIV-1 neutralizing activity. However, addition of the CHR-peptide-based anti-HIV drug, enfuvirtide, to the NHR-specific antibody made this nonneutralizing antibody become neutralizing against infection by divergent HIV-1 strains, including those resistant to enfuvirtide [84]. It is likely that the interaction between enfuvirtide and NHR slowed down the membrane fusion process in a way similar to the effect of lowered temperature, making the NHR accessible to the NHR-specific antibody for mediating inhibition of HIV-1 infection.

Similarly, Richard et al. have used small-molecule CD4-mimetic compounds to induce the conformational change of the HIV-1 Env on HIV-1-infected cells so that these cells become susceptible to ADCC mediated by antibodies in sera from HIV-1-infected patients. Particularly, one of the small-molecule CD4-mimetic compounds could sensitize endogenously infected ex vivo-amplified primary CD4 T cells to ADCC mediated by autologous effector cells and antibodies in the patients' sera. Thus, CD4 mimetics holds the promise of therapeutic utility in preventing and controlling HIV-1 infection [85].

The above findings suggest that a CD4 molecule, CD4 domain, or small-molecule CD4 mimetic can trigger the HIV-1 Env conformation change, resulting in the exposure of some conserved epitopes or region in gp120 or gp41, which can serve as a target for antibodies with a neutralizing effect of ADCC effect, or for peptide-based HIV-1 fusion inhibitors, such as enfuvirtide. These strategies have potential for use in clinics towards a functional HIV cure.

## 4. Conclusion

**Figure 2:**
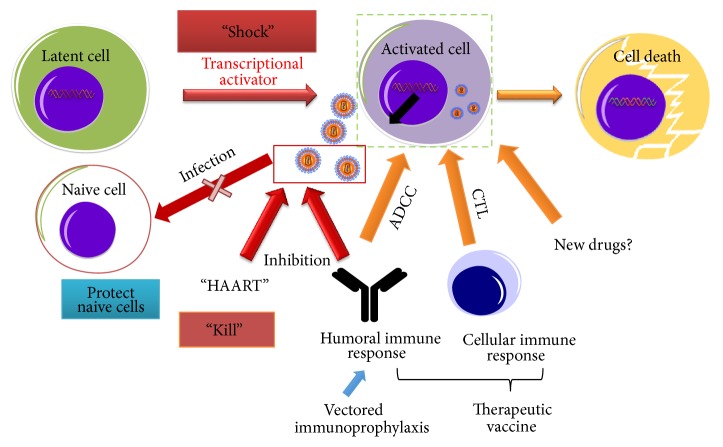
Potential combination of “Shock & Kill,” therapeutic vaccine, and vectored immunoprophylactic strategies to eradicate HIV reservoirs. Specific cytotoxic T lymphocytes (CTL) combined with immunotherapies, such as therapeutic vaccines, broadly neutralizing antibodies, and nonneutralizing antibodies with ADCC effect, may lead to eradication of the latent HIV reservoir.

In this review, we have summarized the advancements in developing strategies for (i) a sterilizing HIV cure and (ii) a functional HIV cure. To achieve a sterilizing HIV cure, three major genome editing technologies, including ZFN, TALENS, and CRISPR-Cas9, have been under development to eliminate the HIV provirus in the latently infected cells. An effective gene therapy strategy is expected to prevent susceptible cells from HIV infection, inspired by the successful cure of HIV infection in the “Berlin patient.” Shock and kill strategy utilizing several well characterized “shock agents” and “kill agents” is still believed to be the most effective way to eradicate latent HIV reservoirs and prevent HIV from rebounding after treatment is stopped (Figure 2). Besides the HIV-specific CTLs, antibody drugs with high ADCC effect seem to be a fruitful developmental direction for the “shock and kill” strategy [58, 86]. To attain functional HIV cure, several highly potent HIV-specific bNAbs have been discovered and developed. These bNAbs have shown excellent in vivo efficacy of functional cure of HIV in SHIV-infected NHPs or HIV-infected patients. The bNAbs or IgG conjugated with CD4 or CD4 domain(s), so-called immunoadhesins, have also displayed substantial in vitro and in vivo efficacies against HIV-1 infection. To solve the problems of high expensiveness to produce antibodies and multiple injections of an antibody, due to its short life, several vector-mediated gene transfer therapies, designated vectored immunoprophylaxis (VIP), have been developed for long-term expression of bNAbs in infected animals or patients. These strategies seem successful in animal models, but there is still a long way for them to go to reach human clinical trials. Development of therapeutic vaccines for a functional HIV cure has faced great challenges over the past 10 years with difficulties of generating effective HIV-specific CTLs, bNAbs, and antibodies with ADCC effects. Nevertheless, the advancement of developing a functional HIV cure seems more rapid and effective than that of a sterilizing HIV cure. The dawn is breaking.

## Figures and Tables

**Table 1:** Reagents to reactivate HIV-1 from latency.

Latency reactivating reagents	Mechanism	Model	Advantage or disadvantage	Reference
Valproic acid (VPA)	HDAC inhibitors	Clinical	Weak and nonspecific	[87]
Butyric acid	HDAC inhibitors	Clinical	Approved by the FDA	[88]
Vorinostat (SAHA/MK0683)	HDAC inhibitors	ACH-2/U1/J-LAT/resting CD4^+^ cells	Approved by the FDA	[39, 89]
Givinostat	HDAC inhibitors	HIV-infected cell lines/Clinical	Superior to VPA	[90]
Panobinostat	HDAC inhibitors	Clinical/HIV-infected cell lines	Lower toxicity	[39, 91]
Apicidin	Histone modification	A10.6 cell line	Synergize with trichostatin A	[92]
Scriptaid	Increasing the acetylation level of histones H3 and H4	Jurkat T cell line	Lower toxicity	[93]
MC1293	HDAC inhibitor	Latency cell lines	Lower toxicity compared to trichostatin A	[47]
M344	HDAC inhibitor	Jurkat T cell	An important role for histone modifications	[94]
As2O3	Affecting the transcription factors and pathways	Jurkat T cell	Synergistically with prostratin or VPA	[48]
OTX015	Activating NF-kappaB	Cell	EC50 value lower than JQ1	[42]
Entinostat (MS275)	Inhibiting HDAC1 and HDAC3	A7 cell	Cytotoxicity lower than SAHA	[95]
Romidepsin	HDAC inhibitor	Clinical/HIV-infected cell lines	Safe	[96]
JQ1	BET inhibitor	Cell lines	A versatile chemical scaffold, binding to its bromodomains	[40]
I-BET151	BET inhibitor	T cells from cART aviremic patients	P-TEFb-releasing agents	[97]
MS417	BET inhibitor	J-Lat cell lines, primary CD4^+^ T cells	Releasing BRD4 from the 5′LTR	[97]
Interleukin (IL)-2	Cytokines and chemokines	HIV-1-infected CD4^+^ T cells	With limited success	
Interleukin (IL)-7	Cytokines and chemokines	Peripheral blood mononuclear cells	Increasing viral production in productively infecting cells without disrupting the latency	[98, 99]
Aza-CdR	DNMT inhibitors	Latently infected cells	An FDA approved drug	[43]
Chaetocin	DNMT inhibitors	Jurkat T cells	Increasing HIV expression without significant toxicity	[100]
HMTi 3-deazaneplanocin A	DNMT inhibitors	Resting CD4^+^ T cells or Jurkat T cells	Targeting HKMT enhancer of Zeste 2	[101]
BIX-01294	DNMT inhibitors	Resting CD4^+^ T cells or Jurkat T cells	Synergistic action, the first HMT inhibitor used to reactivate HIV-1 in vitro	[102]
Ingenol B	PKC activators	J-Lat A1 cell	More potent than prostratin, SAHA and JQ1	[44]
HMBA	P-TEFb activators	Resting CD4^+^ cells	Overcoming barriers to LTR expression	[103]
Disulfiram	Unclassified agents	CD4^+^ cells	Clinical trial	
ZF-VP64	Specific binding to the 5′-LTR promoter	Latently infected cells	Without altering cell proliferation or cell cycle progression	[45]
TALE1-VP64	Transcription activator-like effector	C11 and A10.6 cells	No distribution of cell proliferation or cell cycle	[46]
Dilazep	Nucleoside transport inhibitor	Jurkat T cell	Synergistically reactivated transcription with valproic acid	[49]
